# High Education, Low Returns: Financial Literacy Challenges for African Americans and Hispanics

**DOI:** 10.31586/ojer.2024.1112

**Published:** 2024-11-19

**Authors:** Shervin Assari, Hossein Zare, Amanda Sonnega

**Affiliations:** 1 Marginalized-Related Diminished Returns (MDRs) Research Center, Los Angeles, CA, USA; 2 Department of Family Medicine, Charles R. Drew University of Medicine and Science, Los Angeles, CA, USA; 3 Department of Urban Public Health, Charles R. Drew University of Medicine and Science, Los Angeles, CA, USA; 4 Department of Health Policy and Management, Johns Hopkins Bloomberg School of Public Health, Baltimore, MD, USA; 5 School of Business, University of Maryland Global Campus (UMGC), Adelphi, MD, USA; 6 Institute for Social Research, University of Michigan, Ann Arbor, CA, USA

**Keywords:** Financial Literacy, Educational Attainment, Minorities’ Diminished Returns, Racial Disparities, Hispanic, African American, Ethnic Groups

## Abstract

**Background:**

Education is widely regarded as a key driver of financial literacy, yet racial and ethnic disparities persist. Even among highly educated individuals, African American and Hispanic populations may face challenges in financial literacy, likely due to structural racism and socioeconomic inequalities that diminish the benefits of education. This study examines the relationship between education and financial literacy among African American and Hispanic individuals compared to their White counterparts, focusing on how structural factors contribute to these disparities.

**Objective:**

To determine whether highly educated African American and Hispanic individuals exhibit lower financial literacy compared to similarly educated White individuals and to explore the role of structural factors in explaining these disparities.

**Methods:**

Data from the 2016 Understanding America Study (UAS) were used to evaluate financial literacy among U.S. adults. The sample was stratified by race/ethnicity (African American, Hispanic, and White) and educational attainment. Financial literacy was assessed using standardized financial knowledge tests. Multivariate regression models were employed to investigate the relationship between race/ethnicity, education, and financial literacy, adjusting for socioeconomic factors such as income, employment, and household wealth.

**Results:**

African American and Hispanic individuals with higher levels of education demonstrated significantly lower financial literacy scores than their White peers (p < 0.001). The positive association between education and financial literacy was notably stronger for White individuals than for African American and Hispanic individuals. Further analyses suggest that structural barriers, including systemic discrimination in access to financial resources and disparities in educational quality, contribute to these diminished returns on education for racial and ethnic minorities.

**Conclusion:**

This study highlights persistent financial literacy disparities among highly educated African American and Hispanic individuals, underscoring the limitations of education alone in overcoming structural inequalities. The findings emphasize the need for targeted policies to address systemic barriers that restrict the financial knowledge and opportunities typically associated with higher education for racial and ethnic minority groups.

## 1. Introduction

Financial literacy refers to an individual’s ability to understand and effectively use various financial skills, including personal financial management, budgeting, and investing [1]. It encompasses a broad range of competencies, such as understanding financial concepts like interest rates, inflation, credit scores, and other critical knowledge integral to retirement planning. Financial literacy enables individuals to make informed and effective decisions regarding the use and management of money. Highly financially literate individuals can navigate complex financial products and services, avoid common financial pitfalls, and achieve long-term financial stability [1].

High levels of financial literacy are essential not only for individual well-being but also for broader economic stability. Financial literacy empowers individuals to make sound decisions, such as managing debt, saving for emergencies, investing for retirement, and building wealth. With strong financial literacy, individuals can avoid predatory financial products, reduce the risk of bankruptcy, and improve their credit scores [1]. On a societal level, a financially literate population strengthens the economy, as informed consumers are less likely to default on loans, experience financial stress, or rely heavily on social safety nets. In today’s increasingly complex financial landscape, financial literacy is crucial for managing challenges like student loans, mortgages, and retirement planning—particularly against a backdrop of growing economic inequality and volatile financial markets [1].

Despite the well-established link between education and financial literacy, research shows that African American and Hispanic individuals often derive fewer financial benefits from education than their White counterparts [2–5]. Even at similar levels of education, African American and Hispanic individuals tend to face greater occupational stress, earn lower wages, work in more precarious jobs, and experience harsher workplace conditions [6–8]. According to the theory of Minorities’ Diminished Returns (MDRs), the positive effects of education on various economic and behavioral outcomes are often less pronounced for marginalized racial and ethnic minorities [1, 9–20]. This disparity extends to other areas, including health outcomes and wealth accumulation, where structural inequalities, labor market discrimination, and limited access to high-quality resources undermine the benefits of education for racial and ethnic minorities [21].

Building on the MDRs framework [21], particularly in the context of life course, retirement, and occupational experiences [6–8], this study examines the relationship between educational attainment and financial literacy, focusing on variations by race and ethnicity. We hypothesize that while higher levels of education will generally be associated with greater financial literacy, the strength of this relationship will be weaker for African American and Hispanic individuals, reflecting the diminished returns experienced by these groups due to systemic barriers.

## 2. Methods

The Understanding America Study (UAS) [22–26] is a large, nationally representative, internet-based survey conducted by the University of Southern California (USC). The UAS is designed to gather extensive insights on a wide array of social, economic, and health-related issues across the U.S. population. The study employs probability-based sampling methods, drawing from post-office delivery sequence files to recruit participants. To ensure full inclusivity and representativeness, individuals who do not have internet access are provided with internet-enabled devices, such as tablets, along with internet services, enabling them to participate in the surveys.

The UAS panel includes about 10,000 participants, with nearly 5,000 individuals aged 50 and older. The UAS collects detailed data on numerous domains, including well-being, retirement readiness, cognitive functioning, health behaviors, and personality traits. These surveys are administered regularly—either annually or biennially—to capture longitudinal data on participants. In addition to socioeconomic and behavioral variables, UAS includes health-related metrics to provide continuous assessments of the participants’ health status over time.

The UAS [22–26] provides a rich dataset for examining the relationship between educational attainment and financial literacy, particularly when investigating variations by race and ethnicity. Its comprehensive data collection process allows for the exploration of how different demographic factors, such as age and employment, may moderate the association between education and financial literacy.

For this study, we utilized data from the 2016 wave of the Understanding America Study (UAS), focusing on participants aged 18 to 75 and older. The primary outcome variable was financial literacy, measured on a continuous scale, with higher scores indicating greater financial skills and knowledge essential for retirement preparedness. This measure assessed participants’ understanding of key financial concepts, including financial markets, mortgage rates, savings, pensions, and income stability. A high financial literacy score reflected a strong comprehension of financial mechanisms, such as interest rates, investment strategies, and economic principles.

In addition to financial literacy, participants provided demographic information, including sex, age, marital status, country of birth (U.S.-born vs. non-U.S.-born), current employment status, self-rated health (on a 1–5 scale), and the presence of chronic medical conditions (e.g., heart disease, asthma, diabetes, hypertension). These variables were included as covariates to account for potential confounding effects on financial literacy.

### 2.1. Data Analysis

To explore racial and ethnic differences in financial literacy, initial independent t-tests were conducted to assess variations in financial literacy between Hispanic and non-Hispanic individuals, as well as between White and African American groups. Additionally, t-tests were used to compare age and educational attainment across these racial and ethnic groups. Next, linear regression models were employed to examine the relationship between educational attainment (measured by years of schooling) and the financial literacy, while adjusting for key demographic and health factors such as race, ethnicity, age, sex, employment status, marital status, nativity, self-rated health, and chronic medical conditions. Two models were specified for the analysis: Model 1 included educational attainment, race, ethnicity, and other control variables, without interaction terms. Model 2 incorporated interaction terms between race/ethnicity and years of education to assess whether the relationship between educational attainment and financial literacy varied by race and ethnicity. Results were reported as beta coefficients, along with p-values and 95% confidence intervals (CIs). This analytical approach allowed us to test potential differences in how educational attainment impacts financial literacy across racial and ethnic groups, with a particular focus on the Minorities’ Diminished Returns (MDRs) theory [27–35]. According to MDRs, the effects of educational attainment on outcomes like financial literacy are expected to be less substantial for Hispanic and African American individuals compared to non-Hispanic Whites [9, 10, 14, 16, 18, 21, 36].

### 2.2. Ethical Considerations

The protocol for the Understanding America Study (UAS) was approved by the Institutional Review Board (IRB) at the University of Southern California. All participants provided informed consent prior to participation, and all data were analyzed in an anonymized format to ensure confidentiality.

## 3. Results

### 3.1. Descriptive Statistics, Overall

As shown in Table 1, the study sample consisted of 6,229 participants with a mean age of 48.80 years (SD = 15.55). On average, participants had completed 11.07 years of schooling (SD = 2.28), and the mean number of chronic medical conditions was 1.07 (SD = 1.24). The average self-rated health score was 2.56 (SD = 1.00), and the mean financial literacy score was 9.45 (SD = 3.10). In terms of gender distribution, 43.8% of the participants were men (n = 2,727) and 56.2% were women (n = 3,502). Regarding race, the majority of the sample identified as White (90.6%, n = 5,645), with 9.4% (n = 584) identifying as African American. For ethnicity, 89.7% of participants were non-Hispanic (n = 5,588), while 10.3% (n = 641) identified as Hispanic. The sample included both U.S.-born and immigrant participants, with 94.0% (n = 5,858) being non-immigrants and 6.0% (n = 371) identifying as immigrants. In terms of employment status, 58.5% (n = 3,644) were employed, 41.4% (n = 2,579) were not working, and 6 participants (0.1%) had missing employment data. Regarding marital status, 58.4% (n = 3,637) were married, while 41.6% (n = 2,589) were not married, with a negligible number of participants (n = 3, 0.0%) having missing data on marital status.

### 3.2. Main Effects Model

Table 2 shows the linear regression with financial literacy as the outcome, in the absence of any interaction terms. Years of education is one of the strongest positive determinants of financial literacy (B = 0.526, SE = 0.016, Beta = 0.385, p < 0.001, 95% CI [0.496, 0.557]). Being African American (B = −1.602, SE = 0.118, Beta = −0.150, p < 0.001, 95% CI [−1.832, −1.371]) and being Hispanic (B = −0.908, SE = 0.119, Beta = −0.089, p < 0.001, 95% CI [−1.142, −0.674]) are significantly associated with lower financial literacy. Age is a significant positive predictor of financial literacy (B = 0.048, SE = 0.003, Beta = 0.239, p < 0.001, 95% CI [0.043, 0.053]), while being female is associated with lower financial literacy (B = −1.027, SE = 0.069, Beta = −0.164, p < 0.001, 95% CI [−1.162, −0.892]). Employment status positively predicts financial literacy (B = 0.391, SE = 0.076, Beta = 0.062, p < 0.001, 95% CI [0.242, 0.541]), as does living in a married household (B = 0.448, SE = 0.071, Beta = 0.071, p < 0.001, 95% CI [0.310, 0.587]). However, poorer self-rated health (SRH) is negatively associated with financial literacy (B = −0.263, SE = 0.039, Beta = −0.085, p < 0.001, 95% CI [−0.339, −0.187]). However, being an immigrant shows no significant association with financial literacy (B = 0.190, SE = 0.151, Beta = 0.014, p = 0.210, 95% CI [−0.107, 0.487]) and chronic medical conditions are not significantly related to financial literacy (B = −0.002, SE = 0.034, Beta = −0.001, p = 0.950, 95% CI [−0.069, 0.064]).

### 3.3. Interaction Model

The linear regression analysis with the interaction term (Table 3) examines the predictors of financial literacy. Years of education is a strong positive predictor of financial literacy (B = 0.662, SE = 0.060, Beta = 0.485, t = 10.939, p < 0.001, 95% CI [0.543, 0.780]). Interaction terms show significant negative effects for African American individuals (B = −0.105, SE = 0.053, Beta = −0.131, t = −1.974, p = 0.048, 95% CI [−0.209, −0.001]) and Hispanic individuals (B = −0.157, SE = 0.051, Beta = −0.163, t = −3.099, p = 0.002, 95% CI [−0.257, −0.058]) when considering years of education. However, the interaction between education and immigrant status is not significant (B = −0.009, SE = 0.058, Beta = −0.008, t = −0.161, p = 0.872, 95% CI [−0.123, 0.104]). Age is a significant positive predictor of financial literacy (B = 0.048, SE = 0.003, Beta = 0.239, t = 18.438, p < 0.001, 95% CI [0.043, 0.053]), while being female is associated with lower financial literacy (B = −1.025, SE = 0.069, Beta = −0.164, t = −14.875, p < 0.001, 95% CI [−1.160, −0.890]). Employment status is positively related to financial literacy (B = 0.407, SE = 0.076, Beta = 0.065, t = 5.352, p < 0.001, 95% CI [0.258, 0.556]), as is living in a married household (B = 0.445, SE = 0.071, Beta = 0.071, t = 6.311, p < 0.001, 95% CI [0.307, 0.584]). Chronic medical conditions do not significantly impact financial literacy (B = 0.002, SE = 0.034, Beta = 0.001, t = 0.072, p = 0.943, 95% CI [−0.064, 0.069]). However, poorer self-rated health is negatively associated with financial literacy (B = −0.257, SE = 0.039, Beta = −0.083, t = −6.633, p < 0.001, 95% CI [−0.332, −0.181]).

## 4. Discussion

The primary objective of this study was to investigate the relationship between educational attainment—measured in years of schooling—and financial literacy in a nationally representative sample of U.S. adults. Specifically, we examined whether this relationship varied by race and ethnicity within the framework of Minorities’ Diminished Returns (MDRs). MDRs theory suggests that the financial, economic, cognitive, behavioral, and health benefits of educational attainment are often less pronounced for racial and ethnic minorities compared to non-Hispanic Whites. These diminished returns are attributed to structural marginalization and racialization, which result in racial and ethnic minorities receiving lower-quality education and being more likely to work in less favorable occupations. By comparing the impact of education on financial literacy among African American, Hispanic, and non-Hispanic White individuals, this study aimed to uncover whether disparities in retirement planning and financial literacy could be explained by these differential returns on education across racial and ethnic groups.

Our findings indicate that higher educational attainment is significantly associated with greater financial literacy, emphasizing the pivotal role of schooling in enhancing financial knowledge and skills. However, consistent with the MDRs framework, the results demonstrate that the positive effect of education on financial literacy is significantly weaker for African American and Hispanic individuals compared to non-Hispanic Whites. This discrepancy suggests that even at higher levels of education, African American and Hispanic participants exhibit lower financial literacy than their non-Hispanic White counterparts. These findings highlight the persistent structural barriers and systemic inequalities faced by racial and ethnic minorities, which undermine their ability to fully benefit from educational attainment. This underscores the need for targeted policy interventions to address the root causes of these disparities and ensure that education leads to equitable outcomes for all racial and ethnic groups.

The results of this study support the Minorities’ Diminished Returns (MDRs) theory [21], which posits that the benefits of education are not distributed equally across racial and ethnic groups. In the context of financial literacy, our findings show that while education is a critical determinant, its effects are less pronounced for African American and Hispanic individuals. This suggests that the structural advantages conferred by education, such as higher income, better job opportunities, and improved financial literacy, are not fully accessible to racial and ethnic minorities in the same way they are to non-Hispanic Whites [6–8]. This diminished effect of education aligns with previous research in other areas, such as health outcomes and wealth accumulation, where similar patterns have been observed.

The results of this study support the Minorities’ Diminished Returns (MDRs) theory [21], which posits that the benefits of education and other socioeconomic resources are not distributed equally across racial and ethnic groups. In the context of financial literacy, our findings show that while education is a critical determinant, its effects are less pronounced for African American and Hispanic individuals. This suggests that the structural advantages conferred by education, such as higher income, better job opportunities, and improved financial literacy, are not fully accessible to racial and ethnic minorities in the same way they are to non-Hispanic Whites [6–8]. This diminished effect of education aligns with previous research in other areas, such as health outcomes and wealth accumulation, where similar patterns have been observed.

Minorities’ Diminished Returns (MDRs) refer to the phenomenon where the benefits of socioeconomic factors, like education, wealth, and employment, are reduced for racial and ethnic minorities compared to non-minorities [37–48]. MDRs exist due to structural inequalities and systemic barriers that disproportionately affect marginalized groups. For example, even highly educated African American and Hispanic individuals may face discrimination in the labor market, leading to lower-paying jobs or fewer opportunities for career advancement compared to similarly educated non-Hispanic Whites [21]. Additionally, minorities often attend underfunded schools and live in communities with fewer resources, which may result in a lower quality of education, even when they achieve higher degrees. Historical and ongoing racism, residential segregation, and inequities in the tax system that funds public education further contribute to these disparities. As a result, the social and economic benefits typically associated with higher education do not fully materialize for these groups, as seen in their reduced financial literacy.

Several factors may explain why education has a weaker effect on financial literacy for racial and ethnic minorities. One key factor is the unequal distribution of resources in public schools, which are often funded by local property taxes. This funding model disproportionately affects minority communities, where property values tend to be lower, resulting in less funding for schools. Consequently, even if minority students complete the same number of years of schooling as their White peers, the quality of education they receive may not be equivalent. Additionally, racial and ethnic minorities face greater challenges in the labor market, including discrimination, lower wages, fewer opportunities for promotion, and reduced access to quality retirement benefits, even when their educational credentials are comparable. These barriers may contribute to the weaker relationship between education and financial literacy for minorities, as their education does not translate into the same financial gains as it does for non-Hispanic Whites.

### 4.1. Implications

Our findings carry significant implications for both policy and practical applications. First and foremost, they suggest that improving educational attainment alone will not be enough to ensure equitable financial outcomes for racial and ethnic minorities, particularly in terms of financial security for retirement. Policymakers must address the structural challenges that hinder minorities from converting their educational achievements into higher financial literacy. This could involve reforms aimed at labor market practices, stricter enforcement of anti-discrimination policies, and the provision of targeted financial planning support for minority communities. Furthermore, the disparities revealed by this study emphasize the need for increased investment in minority communities to ensure that the quality of education and access to financial literacy programs are comparable to those offered to non-Hispanic Whites.

The findings hold significant implications for policy-making, as financial literacy plays a crucial role in retirement planning, saving, and making well-informed financial decisions [49–51]. The UAS incorporates a financial literacy measure initially developed for the ALP, addressing limitations in earlier studies that relied on narrow question sets and excluded younger individuals from their samples [52,53]. While some foundational questions from prior financial literacy surveys [54–59] are included, the UAS expands upon these by introducing additional items that assess respondents’ understanding of stocks, bonds, savings accounts, and other financial topics. Evidence suggests that familiarity with specific financial instruments, terms, and concepts is a stronger predictor of time spent on retirement planning than knowledge of fundamental principles such as interest rates, inflation, and risk diversification [55]. Additionally, the survey measures respondents’ confidence in their financial knowledge using a dedicated scale [56].

### 4.2. Limitations

This study has a few limitations that should be considered. First, our analysis was cross-sectional, which limits the ability to establish causality in the relationship between education and financial literacy. Longitudinal analyses are needed to track how changes in education influences changes in financial outcomes over time, particularly as individuals approach retirement. Second, while the study accounted for key demographic variables, unmeasured factors such as early-life socioeconomic status, quality of education, and prior financial literacy may influence the relationship between education and financial outcomes. Additionally, the study relied on self-reported financial literacy measures, which may not fully capture an individual’s financial preparedness or retirement readiness. Future research should incorporate objective indicators, such as savings, assets, and pension contributions, to provide a more comprehensive understanding of financial literacy.

## 5. Conclusion

In summary, this study demonstrates that while educational attainment plays a critical role in shaping financial literacy among U.S. adults, this benefit is not evenly distributed across racial and ethnic groups. African American and Hispanic individuals experience diminished returns on their education compared to non-Hispanic Whites, particularly in terms of financial literacy. These reduced returns are likely the result of structural inequalities and systemic barriers that prevent minorities from fully reaping the financial rewards of their education. The findings emphasize the need for policies that go beyond increasing educational attainment and tackle the broader societal issues that impede racial and ethnic minorities from benefiting fully from their education. Efforts to address disparities in financial literacy should focus on dismantling structural barriers and improving access to quality education, job opportunities, and financial resources in minority communities.

## Figures and Tables

**Table 1.** Descriptive Data Overall (n = 6229)

	Mean	Std. Deviation
Age (Years)	48.80	15.552
Years of Schooling	11.07	2.278
Number of Chronic Medical Conditions	1.07	1.237
Self-Rated Health	2.56	0.999
Financial Literacy	9.45	3.098
	n	%
Sex/Gender		
men	2727	43.8
women	3502	56.2
Race		
White	5645	90.6
African American	584	9.4
Ethnicity		
Non-Hispanic	5588	89.7
Hispanic	641	10.3
Immigrant		
No	5858	94.0
Yes	371	6.0
Working		
No	2579	41.4
Yes	3644	58.5
Missing	6	0.1
Marital Status		
No	2589	41.6
Yes	3637	58.4
Missing	3	0.0

**Table 2.** Summary of Linear regression without interaction

Model	Unstandardized Coefficients		Standardized Coefficients	Sig.	95.0% Confidence Interval for B	
	B	Std. Error	Beta		Lower Bound	Upper Bound
African American	−1.602	.118	−.150	.000	−1.832	−1.371
Hispanic	−.908	.119	−.089	.000	−1.142	−.674
Immigrant	.190	.151	.014	.210	−.107	.487
Age	.048	.003	.239	.000	.043	.053
Female	−1.027	.069	−.164	.000	−1.162	−.892
Working	.391	.076	.062	.000	.242	.541
Married Household	.448	.071	.071	.000	.310	.587
Chronic Medical Conditions	−.002	.034	−.001	.950	−.069	.064
Self-Rated Health (SRH; Poor)	−.263	.039	−.085	.000	−.339	−.187
Education Years	.526	.016	.385	.000	.496	.557

Dependent Variable: Financial Literacy

**Table 3.** Summary of Linear regression with interaction

Model	Unstandardized Coefficients		Standardized Coefficients	Sig.	95.0% Confidence Interval for B	
	B	Std. Error	Beta		Lower Bound	Upper Bound
African American	−.478	.578	−.045	.408	−1.610	.655
Hispanic	.745	.556	.073	.180	−.345	1.835
Immigrant	.233	.661	.017	.725	−1.063	1.528
Age	.048	.003	.239	.000	.043	.053
Female	−1.025	.069	−.164	.000	−1.160	−.890
Working	.407	.076	.065	.000	.258	.556
Married Household	.445	.071	.071	.000	.307	.584
Chronic Medical Conditions	.002	.034	.001	.943	−.064	.069
Self-Rated Health (SRH; Poor)	−.257	.039	−.083	.000	−.332	−.181
Education Years	.662	.060	.485	.000	.543	.780
African American × Education Years	−.105	.053	−.131	.048	−.209	−.001
Hispanic × Education Years	−.157	.051	−.163	.002	−.257	−.058
Immigrant × Education Years	−.009	.058	−.008	.872	−.123	.104

Dependent Variable: Financial Literacy
